# Systematized Linear Porokeratosis With Palmoplantar and Nail Involvement: A Rare Presentation

**DOI:** 10.7759/cureus.103504

**Published:** 2026-02-12

**Authors:** Greeshma Peddireddy, Divya Raviprakash, Leena Dennis Joseph, Adikrishnan Swaminathan

**Affiliations:** 1 Department of Dermatology, Sri Ramachandra Institute of Higher Education and Research, Chennai, IND; 2 Department of General Pathology, Sri Ramachandra Institute of Higher Education and Research, Chennai, IND

**Keywords:** cornoid lamella, nail involvement, palmoplantar involvement, premalignant, rare presentation, systematized linear porokeratosis

## Abstract

Porokeratosis encompasses a group of genetically and clinically heterogenous dermatoses characterized by abnormal epidermal keratinization, with the cornoid lamella being the hallmark histological feature. Linear porokeratosis, a rare variant of porokeratosis, typically manifests early in life, with lesions that follow the lines of Blaschko and carry the highest risk of malignancy transformation. Here, we present a case of systematized linear porokeratosis in a 48-year-old male with childhood onset, notable for the rare co-existence of palmoplantar and nail involvement. This report emphasizes the importance of long-term surveillance in patients with linear porokeratosis due to its malignant potential.

## Introduction

Porokeratosis is an uncommon disorder of epidermal keratinization that results from the clonal expansion of mutated keratinocytes carrying pathogenic gene variants linked to the mevalonate metabolic pathway [1]. Clinically, it is characterized by solitary or multiple atrophic plaques with raised, hyperkeratotic borders, which correspond histopathologically to the cornoid lamella. Linear porokeratosis is a rare variant that manifests segmentally due to embryonic migration of mutated keratinocyte progenitor cells along Blaschko lines. Linear porokeratosis usually presents during infancy or early childhood and has the highest risk of malignant transformation among variants of porokeratosis [2,3]. We report a rare case of systematized linear porokeratosis with palmoplantar and nail involvement, an exceedingly uncommon presentation, highlighting its clinical rarity and diagnostic significance.

## Case presentation

A 48-year-old male from eastern India presented with complaints of skin lesions involving the right side of the body, associated with pruritus and burning sensation, with childhood onset at the age of eight years. Lesions initially appeared over the right axilla and right thigh, which gradually extended linearly to involve the upper and lower extremities, face, neck, and trunk of the same side. He was born from a non-consanguineous marriage, and family history was noncontributory. The patient had not received any treatment prior to this presentation.

**Figure 1:**
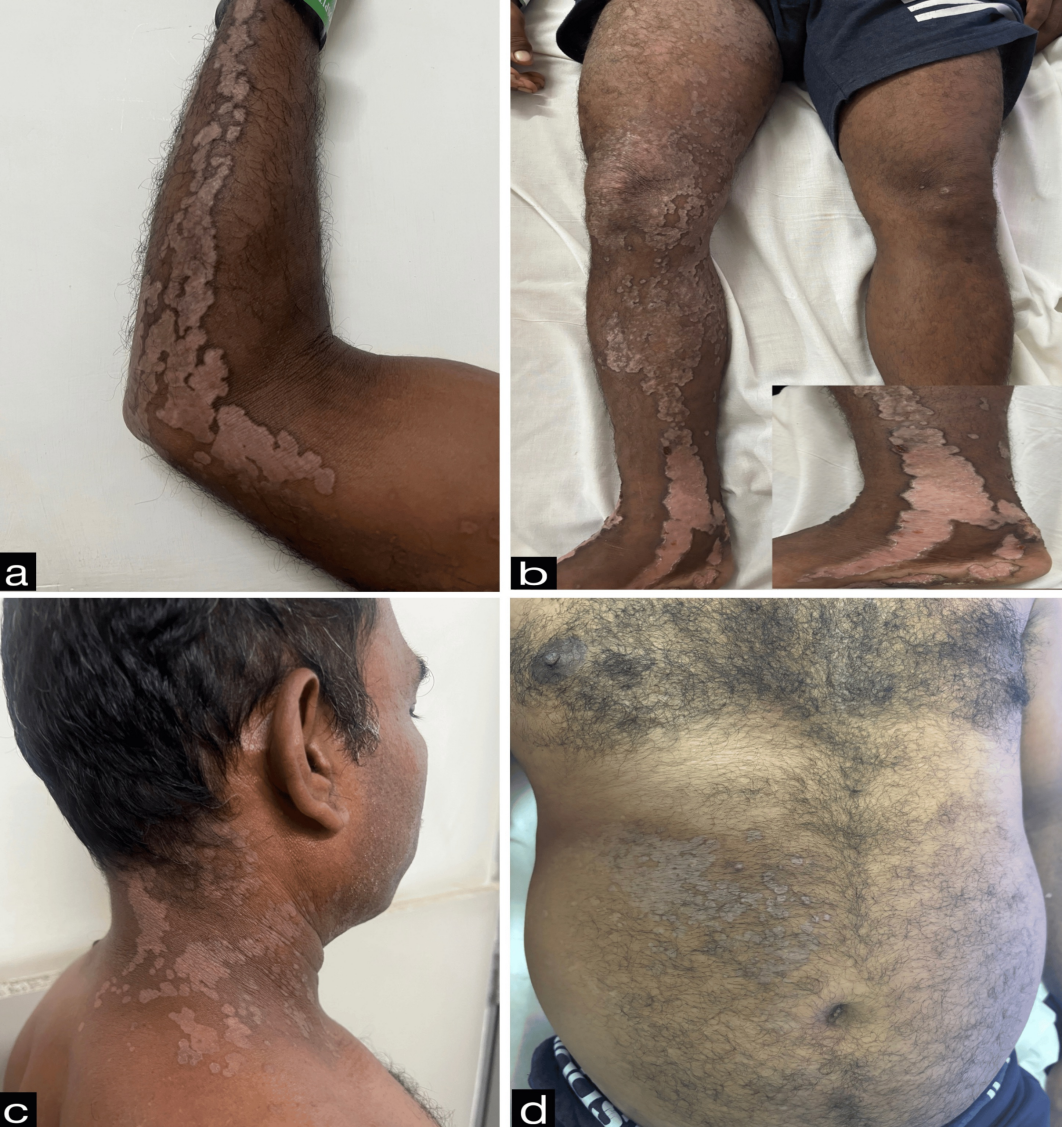
Erythematous to hyperpigmented hyperkeratotic papules and plaques along with atrophic annular plaques having peripheral hyperkeratotic borders on ulnar aspect of the right hand (a), medial aspect of right leg (b), right side of neck (c), and right side of abdomen (d).

**Figure 2:**
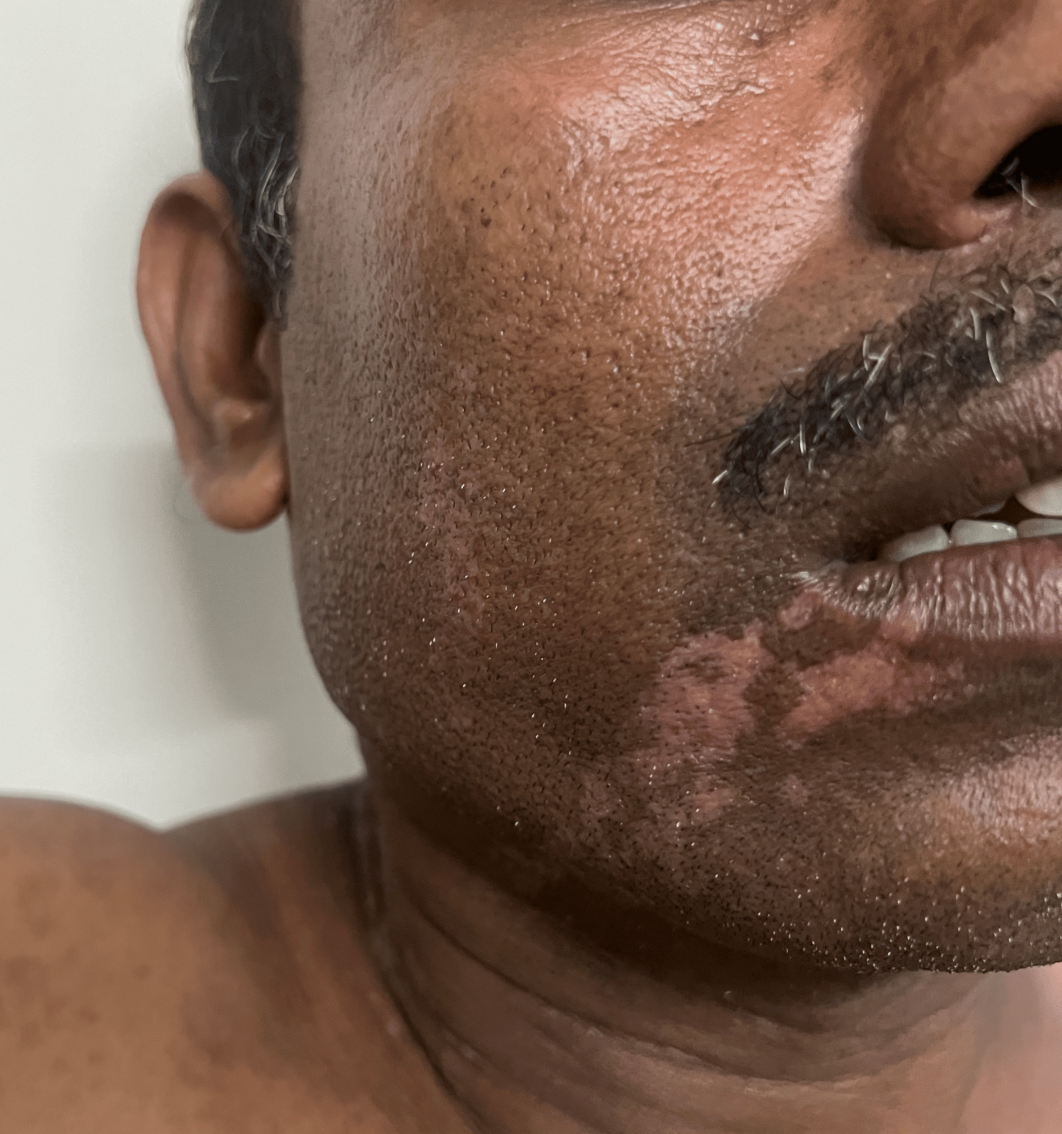
Erythematous papules and plaques along Blaschko lines on the right side of face.

**Figure 3:**
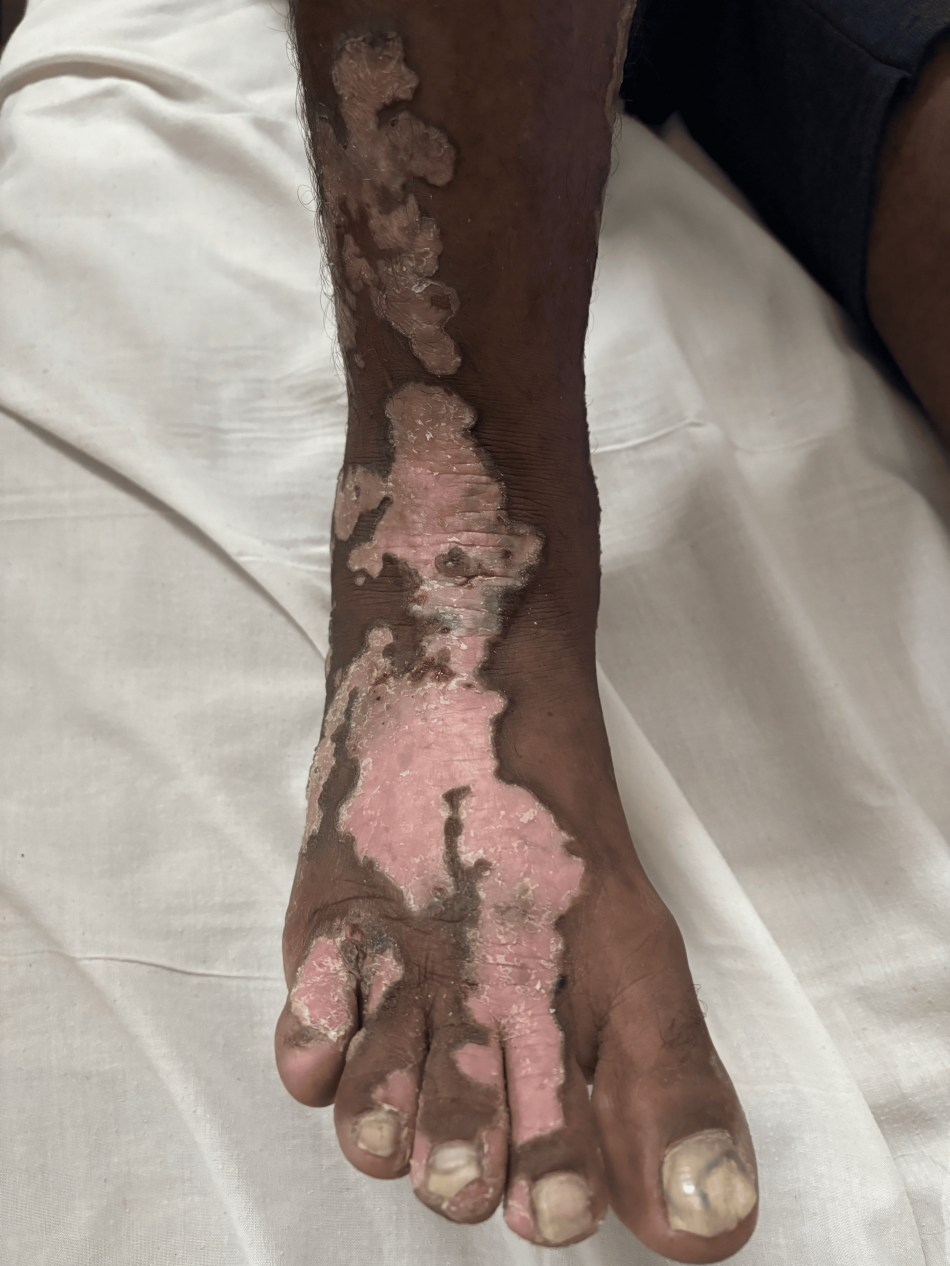
Anonychia of the right fifth toenail.

**Figure 4:**
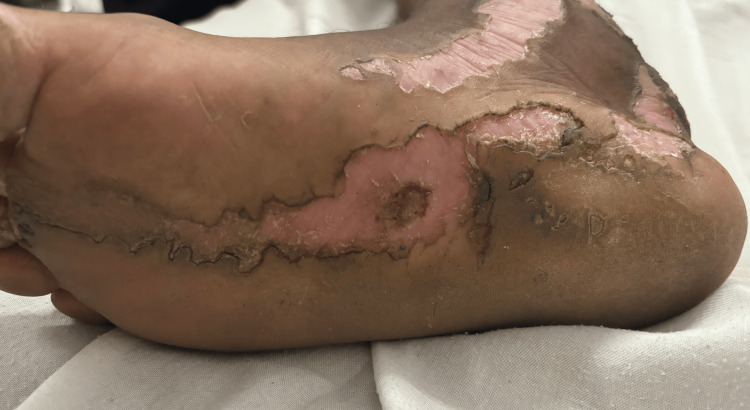
Erythematous atrophic plaques with hyperkeratotic borders demonstrating characteristic longitudinal furrowing over the right sole.

Cutaneous examination revealed unilateral involvement of the right side of the body with linearly arranged discrete to coalescing erythematous and hyperpigmented hyperkeratotic papules, plaques, and annular plaques measuring from 0.5 cm x 0.5 cm to 24 cm x 8 cm. Annular plaques were well-defined, with central hypopigmented to erythematous atrophic centers and peripheral hyperpigmented hyperkeratotic ridge-like borders. A linear streak on the right upper limb extended from the axilla along the ulnar aspect of the arm and forearm to the medial four fingers, involving the palm (Figure 1a). On the right lower limb, lesions followed two linear patterns: one extended from the inguinal crease along the medial and anterior aspects of the thigh, knee, and leg to the medial malleolus, involving the sole (Figure 1b); the other extended from the lateral mid-leg to the dorsum of the foot and the lateral four toes. Similar linear lesions were observed on the right side of the neck, trunk (Figures 1c, 1d), and face (Figure 2), following Blaschko lines. Anonychia of the right fifth toenail was noted (Figure 3). Lesions on the right sole displayed characteristic furrowing (Figure 4). Mucosa and genitalia were spared, and the systemic examination was unremarkable. Clinical differential diagnoses considered were linear porokeratosis, linear discoid lupus erythematosus, and linear verrucous epidermal nevus.

**Figure 5:**
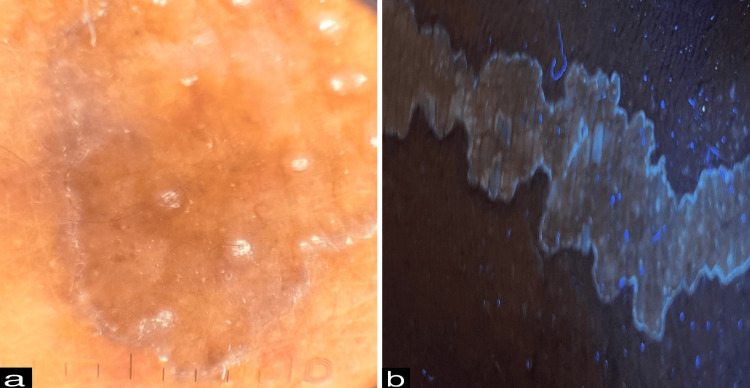
(a) Dermoscopy showed a peripheral white track with a central brown background (10x, polarized mode, DermLite DL5). (b) Ultraviolet-induced fluorescence dermoscopy demonstrated a diamond lace pattern (10x, UV mode, DermLite DL5).

**Figure 6:**
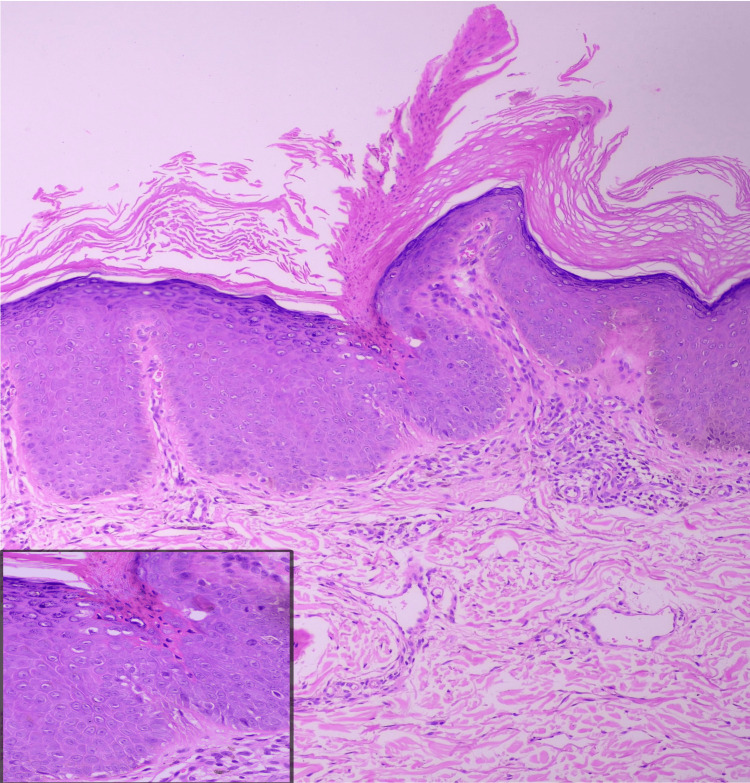
Histopathology revealed cornoid lamella, dyskeratotic keratinocytes (inset), and mild dermal perivascular and periadnexal lymphocytic infiltrates (H&E, 10x).

Dermoscopy revealed a peripheral white track with a central brown background (Figure 5a). Ultraviolet-induced fluorescence dermoscopy demonstrated a characteristic diamond lace pattern (Figure 5b). Histopathological examination of a punch biopsy taken from the hyperkeratotic border showed hyperkeratosis, a vertical column of parakeratotic corneocytes forming cornoid lamella within epidermal invagination, focal absence of the granular layer beneath the cornoid lamella, dyskeratotic keratinocytes, and mild dermal perivascular and periappendageal lymphocytic infiltrates (Figure 6).

Based on clinical, dermoscopic, and histopathological findings, a diagnosis of systematized linear porokeratosis with palmoplantar and nail involvement was established. Complete blood count, fasting lipid profile, liver function test, and lactate dehydrogenase levels were within reference ranges. The patient was initiated on 25 mg of capsule acitretin daily, topical calcipotriol 0.0003% ointment, and advised strict photoprotection. The patient was counseled regarding the malignant potential of the condition and advised regular follow-up every six months.

## Discussion

The term "porokeratosis" was first introduced by Mibelli in 1893 [1]. Porokeratosis is now recognized as a disorder driven by germline heterozygous loss-of-function mutations in mevalonate pathway genes (MVK, PMVK, MVD, and FDPS), followed by post-zygotic/acquired second-hit loss-of-function mutation in lesional skin, which results in biallelic loss and clonal keratinocyte expansion, forming characteristic cornoid lamella. The timing of the second event likely accounts for heterogeneity in onset, extent, and distribution of lesions. Disruption of the mevalonate pathway results in cholesterol deficiency and accumulation of toxic intermediates, impairing epidermal barrier function, rendering keratinocytes more susceptible to pro-apoptotic stimuli, and potentially increasing the risk of carcinogenesis [1,3-5].

Primary lesions include brown-colored, hyperkeratotic papules, which gradually spread centrifugally to form irregular, well-defined annular plaques, which are distinguished by a characteristic longitudinal furrow, a peripheral hyperkeratotic ridge-like border, and an atrophic center associated with anhidrosis and alopecia [1,2,6].

Histopathologically, porokeratosis is defined by the presence of cornoid lamella at the peripheral hyperkeratotic border. The cornoid lamella appears as a tightly packed, thin, vertical column of parakeratotic keratinocytes within a thickened, orthokeratotic stratum corneum and is situated in a shallow epidermal invagination with focal absence of the granular layer. Occasional dyskeratotic and vacuolated keratinocytes may be seen in the underlying spinous and basal layers, accompanied by a moderately dense inflammatory infiltrate in the subjacent papillary dermis [1,2].

On dermoscopy, a well-demarcated, white-colored rim is seen at the periphery, with the center showing dotted vessels or blue-grey dots and granules depending on disease progression [7].

Clinical variants include porokeratosis of Mibelli, linear porokeratosis, punctate porokeratosis, genital porokeratosis, disseminated superficial actinic porokeratosis, disseminated superficial porokeratosis, and disseminated palmoplantar porokeratosis [1,2].

Linear porokeratosis is a rare premalignant variant that may present as localized, zosteriform, systematized, or generalized disease [3]. In linear porokeratosis, an early embryonic second hit produces a large Blaschkoid clone that persists lifelong, which may explain the substantially higher incidence of malignant transformation, reported to be approximately 20% [2,4,8]. Long-term surveillance is therefore essential. To the best of our knowledge, systematized linear porokeratosis with simultaneous palmoplantar and nail involvement is an extremely rare presentation and has been reported only once previously [9].

Therapeutic options include topical vitamin D analogs, retinoids, 5-fluorouracil, imiquimod, and systemic retinoids, all of which act by downregulating abnormal clonal keratinocyte proliferation. Recently, topical cholesterol/lovastatin has emerged as a promising therapy, which replaces cholesterol deficiency and prevents the accumulation of toxic metabolites. Procedural modalities include radiosurgery, cryotherapy, shave excision, surgical excision, and photodynamic therapy for selected cases [1,2,10].

## Conclusions

Linear porokeratosis is a rare premalignant entity that is often underdiagnosed due to its clinical resemblance to other linear dermatoses. Our case report highlights the systematized variant of linear porokeratosis and its unusual presentation in the form of simultaneous palmoplantar and nail involvement. Dermoscopy in conjunction with histopathology facilitates early and accurate diagnosis. Early recognition, patient counselling, and vigilant long-term surveillance are crucial to mitigate the risk of malignancy transformation associated with linear porokeratosis. This report adds to the limited existing literature and reinforces the need for heightened clinical awareness and vigilant monitoring in patients with linear porokeratosis.
